# Nontuberculous scrofuloderma with sweet syndrome leading to anti-interferon-γ autoantibody-associated adult-onset immunodeficiency: A case report

**DOI:** 10.1016/j.jdcr.2025.05.029

**Published:** 2025-06-16

**Authors:** Nutpeera Nutthapan

**Affiliations:** Division of Dermatology, Department of Internal Medicine, Sisaket Hospital, Sisaket, Thailand

**Keywords:** anti-interferon-γ autoantibody-associated adult-onset immunodeficiency, immunodeficiency syndrome, *Mycobacterium abscessus*, nontuberculous scrofuloderma, Sweet syndrome

## Introduction

Scrofuloderma is a contiguous cutaneous mycobacterial infection that spreads from the infected internal structures such as lymph nodes, bones, joints, and testes.^1^ The clinical presentation begins with subcutaneous nodules, which eventually rupture, leading to the formation of fistulas and ulcers.^1^ Most of the cases are caused by *Mycobacterium tuberculosis*.^1^ However, a minority are attributed to nontuberculous mycobacteria (NTM).^2^ Cutaneous NTM infections can occur in individuals regardless of immune status, but disseminated disease may indicate an underlying immunodeficiency.^3^ Aside from HIV infection, severe or disseminated cutaneous NTM infection in adults potentially indicates other acquired adult-onset immunodeficiency syndromes, like anti-interferon-γ autoantibody (AIGA)–associated immunodeficiency.^3^ Interestingly, Sweet syndrome (SS) has been reported in association with both NTM infection and AIGA-associated immunodeficiency.^3^^,^^4^ This report describes a rare case of nontuberculous scrofuloderma accompanied by SS, which finally led to a diagnosis of AIGA-associated immunodeficiency.

## Case report

**Fig 1:**
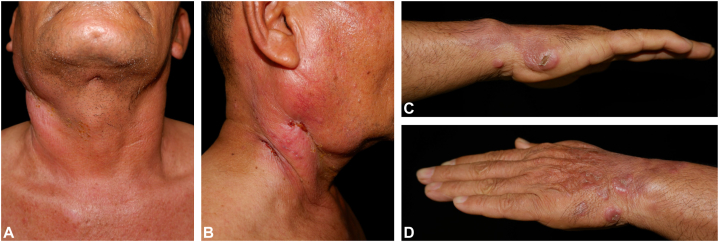
Clinical images. **(A, B)** Ill-defined, indurated, edematous, erythematous plaque with small pustules and sinus tracts on the right side of the neck. **(C, D)** Multiple erythematous papules and nodules on both hands.

A previously healthy 60-year-old man presented with a progressive painful erythematous plaque on the right side of his neck that had developed over 2 months and eventually ulcerated. A month later, he noticed multiple painless erythematous nodules on both hands. He did not recall any history of trauma or constitutional symptoms. Six months earlier, he had experienced similar symptoms on the right side of his neck and visited a local hospital, where he was diagnosed with tuberculous lymphadenitis and received 6 months of medical treatment. His symptoms had completely resolved at that time. On physical examination, there was an ill-defined, indurated, edematous, erythematous plaque with pustules and sinus tracts on the right side of his neck (Fig 1, *A* and *B*), along with multiple erythematous papules and nodules on both hands (Fig 1, *C* and *D*). Additionally, cervical lymphadenopathy was noted.

**Fig 2:**
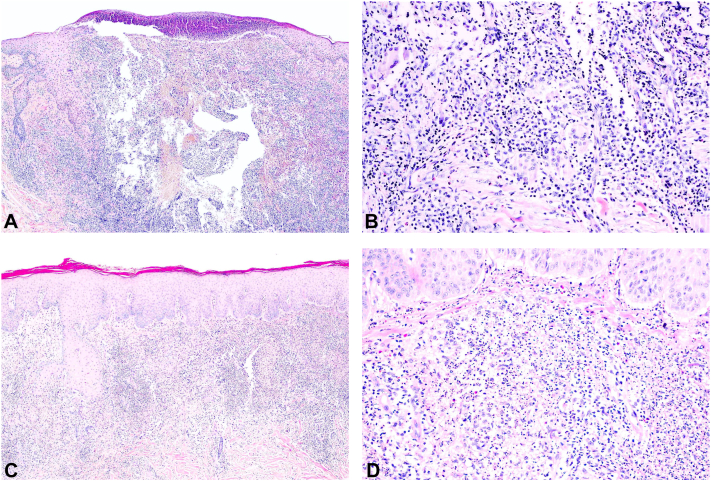
Histopathology from the lesion on the neck. **(A, B)** Tuberculoid granuloma with ulcerated epidermis and pustule. Histopathology from the lesion on the hand. **(C, D)** Diffuse neutrophilic infiltration in the upper dermis without vasculitis (original magnification, hematoxylin and eosin. **A,** ×40; **B,** ×100; **C,** ×40; **D,** ×100).

His laboratory examinations revealed leukocytosis (16,200 cells/μL) with neutrophil predominance in the complete blood count, while other blood chemistries were unremarkable. His serum anti-HIV test was negative. Incisional biopsies were performed on the plaque on his right neck, the nodule on his right hand, and the right cervical lymph node. The pathologic diagnosis and mycobacterial profiles are summarized in Table I. The biopsy of the plaque on his right neck revealed granulomatous dermatitis (Fig 2, *A* and *B*), while the biopsy of the nodule on his right hand showed diffuse neutrophilic infiltration without vasculitis (Fig 2, *C* and *D*). *Mycobacterium abscessus* was detected from the cervical lymph node culture identification. A computed tomography scan showed multiple axillary and intra-abdominal lymphadenopathy. Given the diagnosis of disseminated NTM infection and the negative anti-HIV test, serum testing for anti-interferon-gamma autoantibodies was conducted and returned a positive result.

**Table I:** Pathologic diagnosis and mycobacterial profiles from multiple sites

Site of biopsy	Pathologic diagnosis	AFB stain	PCR for MTB	Mycobacterial culture
Plaque on the right side of the neck	Granulomatous dermatitis	Negative	Negative	No growth
Nodule on the right hand	Sweet syndrome	Negative	Negative	No growth
Right cervical lymph node	Acute lymphadenitis	Negative	Negative	*Mycobacterium abscessus*

*AFB*, Acid-fast bacilli; *MTB*, *Mycobacterium tuberculosis*; *PCR*, polymerase chain reaction.

**Fig 3:**
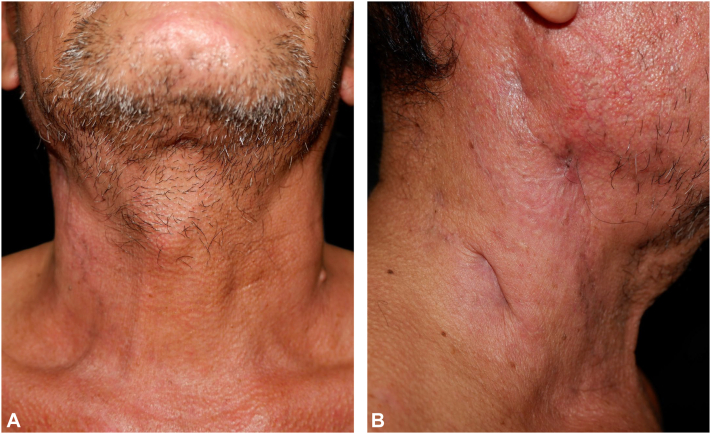
**A** and **B,** Clinical images of the improved lesion on the neck.

Regarding the treatment, the patient received intravenous tigecycline (50 mg every 12 hours for 2 weeks), imipenem (1 g every 12 hours), and amikacin (750 mg every 24 hours), resulting in marked clinical improvement (Fig 3, *A* and *B*). The cervical lymphadenopathy also resolved. For the management of SS, oral colchicine and topical corticosteroids were given, resulting in complete clinical remission. Because the patient experienced a second episode of infection within 6 months, rituximab was scheduled to be administered after the resolution of the infection.

## Discussion

This report presents a rare case of nontuberculous scrofuloderma caused by *Mycobacterium abscessus* in association with SS, which eventually led to the diagnosis of AIGA-associated immunodeficiency. Other reported species of NTM that cause scrofuloderma-like presentations include *Mycobacterium scrofulaceum*, *Mycobacterium kansasii*, *Mycobacterium intracellulare*, and *Mycobacterium chelonae*.^2^ Clinical presentations of cutaneous NTM are typically nonspecific and may manifest as papules, plaques, cellulitis-like lesions, sporotrichoid nodules, panniculitis, ulcers, or sinus tracts.^5^ *Mycobacterium abscessus* is considered the most pathogenic species and a leading cause of skin infections among rapidly growing mycobacteria.^5^ Its incidence has been rising, largely due to contamination in water sources and cosmeceutical products.^5^ Furthermore, *Mycobacterium abscessus* has shown a high prevalence in patients with AIGA-associated immunodeficiency,^6^ which likely explains the causative organism in this case. AIGA-associated immunodeficiency predisposes individuals to disseminated NTM infections.^6^^,^^7^ However, its impact on the cutaneous manifestations of the NTM infections remains unknown.

SS is associated with various conditions, including NTM infection, particularly in cases of disseminated disease, and occurs in approximately 40% of individuals with AIGA-associated immunodeficiency.^4^^,^^7^ In the context of AIGA-associated immunodeficiency, SS is almost always linked to active opportunistic infections, especially NTM infection.^4^ The presence of lymphadenopathy and leukocytosis in SS raises suspicion for AIGA-associated immunodeficiency.^7^ The pustular morphology of SS remains an inconclusive predictor of AIGA-associated immunodeficiency.^7^^,^^8^ One small study found that all patients with NTM infection and concomitant SS were diagnosed with AIGA-associated immunodeficiency.^8^

Treatment for any infections in the AIGA-associated immunodeficiency setting is challenging due to the high rate of disease recurrence.^6^ Until now, there is no definite treatment for AIGA-associated immunodeficiency.^6^ The primary aim of the treatment is to decrease the AIGA level since its level is strongly correlated with disease activities.^9^ Rituximab and intravenous cyclophosphamide significantly reduce the AIGA level and improve clinical outcomes.^6^^,^^9^ However, the resurgence of the AIGA level and infections are the main problems regarding the treatment.^9^ The patients might need multiple courses of treatment and careful observation for infections.^6^^,^^9^ Recently, a regimen of bortezomib, a peptide-based proteasome inhibitor, followed by oral cyclophosphamide was employed for the treatment of AIGA-associated immunodeficiency.^9^ The result showed no significant decrease in the AIGA level due to the lack of effect on plasma cell progenitors.^9^ For that reason, combining other agents targeting the plasma cell progenitors, such as rituximab and daratumumab, may be beneficial.^9^

In conclusion, this report highlights the potential for NTM infection to mimic cutaneous tuberculosis. Cutaneous NTM infection in conjunction with SS may serve as a clue to AIGA-associated immunodeficiency after excluding other possible causes of immunodeficiency, particularly HIV infection. Rituximab and cyclophosphamide can be beneficial adjunctive therapies, in addition to antibiotics, for managing infections in the setting of AIGA-associated immunodeficiency. However, recurrent infections are common, often necessitating multiple treatment sessions. Future studies are required to comprehend the impact of AIGA-associated immunodeficiency on the presentation of NTM infection and to explore additional sustainable therapeutic options.

## Conflicts of interest

None disclosed.
